# Relationship of Neutrophil-to-Lymphocyte and Platelet-to-Lymphocyte Ratio With Visual Acuity After Surgical Repair of Open Globe Injury

**DOI:** 10.3389/fmed.2021.697585

**Published:** 2021-11-22

**Authors:** Karim Mohamed-Noriega, Alan B. Treviño-Herrera, Jibran Mohamed-Noriega, Braulio H. Velasco-Sepúlveda, Víctor A. Martínez-Pacheco, Dante A. Guevara-Villarreal, Delia L. Rodríguez-Medellín, Anna G. Sepúlveda-Salinas, Gerardo Villarreal-Méndez, Jesús H. González-Cortés, Rodrigo E. Elizondo-Omaña, Santos Guzmán-López, Jesús Mohamed-Hamsho

**Affiliations:** ^1^Department of Ophthalmology, University Hospital and Faculty of Medicine, Autonomous University of Nuevo León, Monterrey, Mexico; ^2^Department of Human Anatomy, Faculty of Medicine, Autonomous University of Nuevo León, Monterrey, Mexico

**Keywords:** prognostic, platelet lymphocyte ratio (PLR), neutrophil lymphocyte ratio (NLR), severe visual impairment, ocular injury, ocular trauma, open globe injury (OGI)

## Abstract

**Purpose:** To assess the relationship and prognostic value of the neutrophil-to-lymphocyte ratio (NLR) and platelet-to-lymphocyte ratio (PLR) with poor final best-corrected visual acuity (BCVA) after surgical repair of open globe injuries (OGI) in adults.

**Design:** Retrospective analysis of data from an ongoing prospective cohort of consecutive patients.

**Methods:** In a tertiary university hospital, 197 eyes of 197 patients were included between 2013 and 2017. NLR and PLR were obtained from pre-operative blood tests to analyze its relationship with poor final BCVA.

**Results:** Severe visual impairment (SVI) was defined as ≤20/200, and was observed in 96 (48.7%) patients after surgical repair of OGI. SVI patients had higher NLR (7.4 ± 6.6 vs. 4.0 ± 3.2, *p* < 0.001), and PLR (167 ± 92 vs. 139 ± 64; *p* = 0.021) than non-SVI. NLR ≥ 3.47 and PLR ≥ 112.2 were the best cut-off values for SVI, were univariate risk factors for SVI, and had sensitivity: 69.0, 71.4, and specificity: 63.6, 44.8, respectively. In multivariate analysis, only OTS, athalamia, and hyphema remained as risk factors. NLR had significant correlation with ocular trauma score (OTS) (*r* = −0.389, *p* < 0.001) and final BCVA (*r* = 0.345, *p* < 0.001).

**Limitations:** Simultaneous trauma in other parts of the body that could influence the laboratory findings.

**Conclusion:** Patients with SVI after a repaired OGI had increased pre-operative NLR and PLR levels. High NLR and PLR are risk factors for SVI in univariate analysis. It is confirmed that low OTS is a risk factor for SVI. High NLR and PLR could be used as a prognostic tool to identify patients at higher risk for SVI after repair of OGI.

## Introduction

Open globe injury (OGI) is defined as a full-thickness wound of the eyewall and represents a vision-threatening ocular injury (1, 2). Global estimations indicate that a total of 1.6 million cases of blindness are due to eye injuries, 2.3 million people with low vision are due to ocular injuries and 19 million cases of monocular blindness are due to eye injuries (3). The importance lies in the fact eye injuries, apart from their visual impact, generate work absenteeism, high cost in health care systems, and a serious impact on the quality of life. Being a preventable problem, there is much to be done to avoid this situation.

Risk factors associated with poor final visual acuity (VA) includes poor initial VA, globe rupture, zone 3 injury, the posterior extension of the wound to rectus insertion, presence of relative afferent pupillary defect (RAPD), vitreoretinal trauma, hyphema, cataract, vitreous loss, and low ocular trauma score (OTS) (4–8). In most cases, the diagnosis of OGI is clinical, however, there are different diagnostic methods such as ultrasonography (US) and computed tomography (CT) that can be of aid when the slit-lamp examination or the evaluation of the fundus are not possible (9). In addition, CT may be useful in the search for orbital fractures, for intraocular foreign bodies (IOFB), and as an aid if an occult OGI is suspected. Likewise, different CT scans findings have been associated with poor visual prognosis, helping in the council of patients (10–12). On the other hand, US and ultrasonic biomicroscopy (UBM) are safe and economical methods that can provide valuable information in case of media opacity for the surgical plan elaboration and as predictors for final vision. However, they are operator dependent, and there is a concern of prolapse of the intraocular tissues, so it should be indicated with caution (13, 14).

The counts of white blood cell (WBC), neutrophil (NEU), and lymphocyte (LYM) as well as the neutrophil-to-lymphocyte ratio (NLR), and the platelet-to-lymphocyte ratio (PLR) are widely used as blood biomarkers associated with inflammation. The NLR and PLR have been used in cardiovascular diseases to predict death and myocardial infarction (15–20), and have been related to inflammatory activity in rheumatologic diseases (21, 22). They have been recognized as indicators of poor prognostic for survival in many solid cancers (23, 24) and renal diseases (25).

The NLR and PLR have been also associated with ocular conditions, such as age-related macular degeneration (26–29), idiopathic acute anterior uveitis (30), diabetic retinopathy (31, 32), keratoconus (33), dry eye disease (34, 35), primary open-angle glaucoma (36), and pseudoexfoliation syndrome (37, 38). However, their relationship with the final VA after a surgical repair of OGI has not been studied. The current study aims to assess the relationship and prognostic value of pre-operative NLR and PLR with poor final VA after repaired OGI.

## Methods

Data were retrospectively analyzed from an ongoing prospective cohort that included all consecutive patients with OGI that had primary repair between January 2013 and December 2017 at the ophthalmology department of the University Hospital from the Faculty of Medicine of the Autonomous University of Nuevo León (UANL), a tertiary care university hospital in Monterrey, Mexico. Patients younger than 18 years of age, primary evisceration or enucleation, secondary OGI repairs, and patients operated elsewhere were excluded. The study received institutional ethics committee approval and was conducted following good clinical practices and the declaration of Helsinki. All patients read and provided written informed consent to participate.

All patients underwent a complete ophthalmic evaluation at presentation and in follow-up visits, including pupillary reflexes, and RAPD. Best-corrected visual acuity (BCVA) was performed with a Snellen chart either with subjective refraction or pinhole. The length and zone of the wound were recorded according to the classification of the Ocular Trauma Classification Group (39). Total raw points of the OTS were calculated using the variables of initial BCVA, globe rupture, endophthalmitis, perforating injury, retinal detachment, and RAPD (6). The type of injury was categorized according to the standardized classification of ocular trauma in globe rupture, penetrating injury, perforating injury, or intraocular foreign body (40, 41). The mechanism of injury was recorded and a detailed examination of the anterior and posterior segment was included. Corneal trauma was defined as follows: central corneal trauma when it occurred in the central 3 mm zone, paracentral when it occurred outside the central zone and inside 8 mm diameter, and peripheral when it occurred outside the paracentral zone and up to the limbus. Total corneal trauma was defined when all 3 zones were involved. Several time intervals relative to the accident were recorded. Patients were divided for analysis based on final visual acuity as patients with severe visual impairment (SVI) or non-SVI (NSVI). Patients with SVI were defined as having a final BCVA of 20/200 or worse in the last visit. Final BCVA better than 20/200 were considered non-SVI (NSVI) (42). Patients were further divided according to the presence or absence of systemic comorbidities and substance use.

Blood tests were performed on all patients before surgery. The blood samples were assessed by flow cytometry using a CELL-DYN® Ruby™ (Abbott Laboratories). All the absolute parameters of the blood tests were included, as well as glucose. The NLR was calculated by dividing neutrophils (NEU) by lymphocytes (LYM) and the PLR was calculated by dividing platelets (PTL) by LYM. The mixed monocyte-basophil-eosinophil (MXD) was calculated by the sum of monocytes (MON), basophils (BAS), and eosinophils (EOS).

Descriptive and inferential statistical analyses were performed. The Chi-square statistics, the *T*-test, and the U-Mann Whitney-test were used for univariate associations. Correlation analysis was performed with Spearman's correlation coefficient. Receiver-operating characteristic (ROC) curve was used for determining cut-off values for NLR and PLR to distinguish between SVI and NSVI, chosen based on the maximal Younden's Index. A linear regression model was used for univariate and multivariate logistic analysis. All variables of known clinical relevance whose univariate analysis resulted in a *p*-value ≤ 0.001 and with a high OR were considered for multivariable analysis. The odds ratio (OR) and 95% confidence interval (CI) for each independent variable were calculated from the same model. Statistical significance was considered when *p* ≤ 0.05. The statistical analysis was performed using Microsoft Office Excel 2013 and SPSS (IBM Corp. Released 2011. IBM SPSS Statistics for Windows, Version 20.0. Armonk, NY: IBM Corp).

## Results

Of the 271 patients included in the cohort, 66 patients younger than 18 years old, and 8 with missing worksheets were excluded; 197 eyes of 197 adult patients were included for analysis, 96 (48.7%) developed SVI. Patients with SVI were significantly older (39.5 ± 15.4 vs. 35.3 ± 12.9; *p* = 0.043). Most of the patients were male in SVI (89.6%) as well as in NSVI (90.1%) groups (*p* = 1.000). The median (IQR) follow-up time was significantly longer in NSVI [90 (129) days] than in SVI [30 (80) days] patients (*p* < 0.001). Time intervals between accident and consultation, surgery or blood sample analysis were similar between patients with SVI, and NSVI (*p* > 0.05). Illegal substance use and the presence of systemic comorbidities were significantly more frequent in patients with SVI (*p* < 0.05). The mechanisms of injury had a significantly different distribution between groups; polytrauma, fist/kick and blunt object were observed more often in patients with SVI, whereas metallic objects were observed more often in patients with NSVI. Detailed demographic characteristics, time intervals, and systemic comorbidities are shown in Table 1.

**Table 1:** Demographics, time intervals and mechanism of injury in SVI and NSVI patients.

**Characteristics**	**All**	**SVI**	**NSVI**	***P*=**
	**(*n* = 197)**	**(*n* = 96)**	**(*n* = 101)**	
Gender, Men (*n*, %)	177 (89.8)	86 (89.6)	91 (90.1)	1.000
Age, (mean ± SD) years	37.3 ± 14.3	39.5 ± 15.4	35.3 ± 12.9	**0.043**
Cases <50 yo (*n*, %)	163 (82.7)	75 (78.1)	88 (87.1)	0.131
Laterality, left eye (*n*, %)	109 (55.3)	54 (56.3)	55 (54.5)	0.886
Follow-up (median, IQR) days	75, 120	30, 80	90, 129	**<0.001**
**Accident-consultation interval**				
Mean hours ± SD	29.0 ± 60.6	28.2 ± 44.9	29.7 ± 72.3	0.872
<24 h (*n*, %)	116 (70.7)	50 (64.1)	66 (76.7)	0.087
**Accident-surgery interval**				
Mean hours ± SD	67.7 ± 95.8	60.7 ± 51.6	73.8 ± 121.5	0.358
<24 h (*n*, %)	29 (17.8)	13 (17.3)	16 (18.2)	1.000
**Accident-blood sample interval**				
Mean hours ± SD	46.9 ± 84.3	44.4 ± 75.5	49.2 ± 92.3	0.729
<24 h (*n*, %)	86 (59.3)	42 (60.0)	44 (58.7)	1.000
One or more systemic comorbidities (*n*, %)	100 (50.8)	58 (60.4)	42 (41.6)	**0.010**
**Systemic diseases**				
Systemic hypertension (*n*, %)	14 (7.7)	10 (11.6)	4 (4.1)	0.092
Diabetes mellitus (*n*, %)	8 (4.4)	5 (5.8)	3 (3.1)	0.478
Other systemic diseases (*n*, %)	5 (2.5)	2 (2.1)	3 (3.0)	1.000
**Substance use**				
Tobacco (*n*, %)	55 (30.2)	26 (30.2)	29 (30.2)	1.000
Alcohol (*n*, %)	44 (24.2)	25 (29.1)	19 (19.8)	0.167
Illegal substances (*n*, %)	16 (8.8)	12 (14.0)	4 (4.2)	**0.033**
**Mechanism of injury**				
Metallic object (*n*, %)	75 (39.1)	18 (19.6)	57 (57.0)	**<0.001**
Stone (*n*, %)	19 (9.9)	11 (12.0)	8 (8.0)	0.469
Glass (*n*, %)	18 (9.4)	8 (8.7)	10 (10.0)	0.808
Polytrauma (*n*, %)	17 (8.9)	14 (15.2)	3 (3.0)	**0.004**
Fist/kick (*n*, %)	14 (7.3)	12 (13.0)	2 (2.0)	**0.004**
Blunt object (*n*, %)	11 (5.7)	10 (10.9)	1 (1.0)	**0.004**
Branch/stick (*n*, %)	13 (6.8)	4 (4.3)	9 (9.0)	0.256
Knife (*n*, %)	4 (2.1)	2 (2.2)	2 (2.0)	1.000
Explosives (*n*, %)	3 (1.6)	3 (3.3)	0 (0.0)	0.108
Others (*n*, %)	18 (9.2)	10 (10.9)	8 (8.0)	0.622

*IQR, Interquartile range; SVI, Severe visually impaired; NSVI, Not-severe visually impaired; OGI, Open globe injury*.

*Bold means p < 0.05. Percentages are relative to the number of patients with available data*.

**Figure 1:**
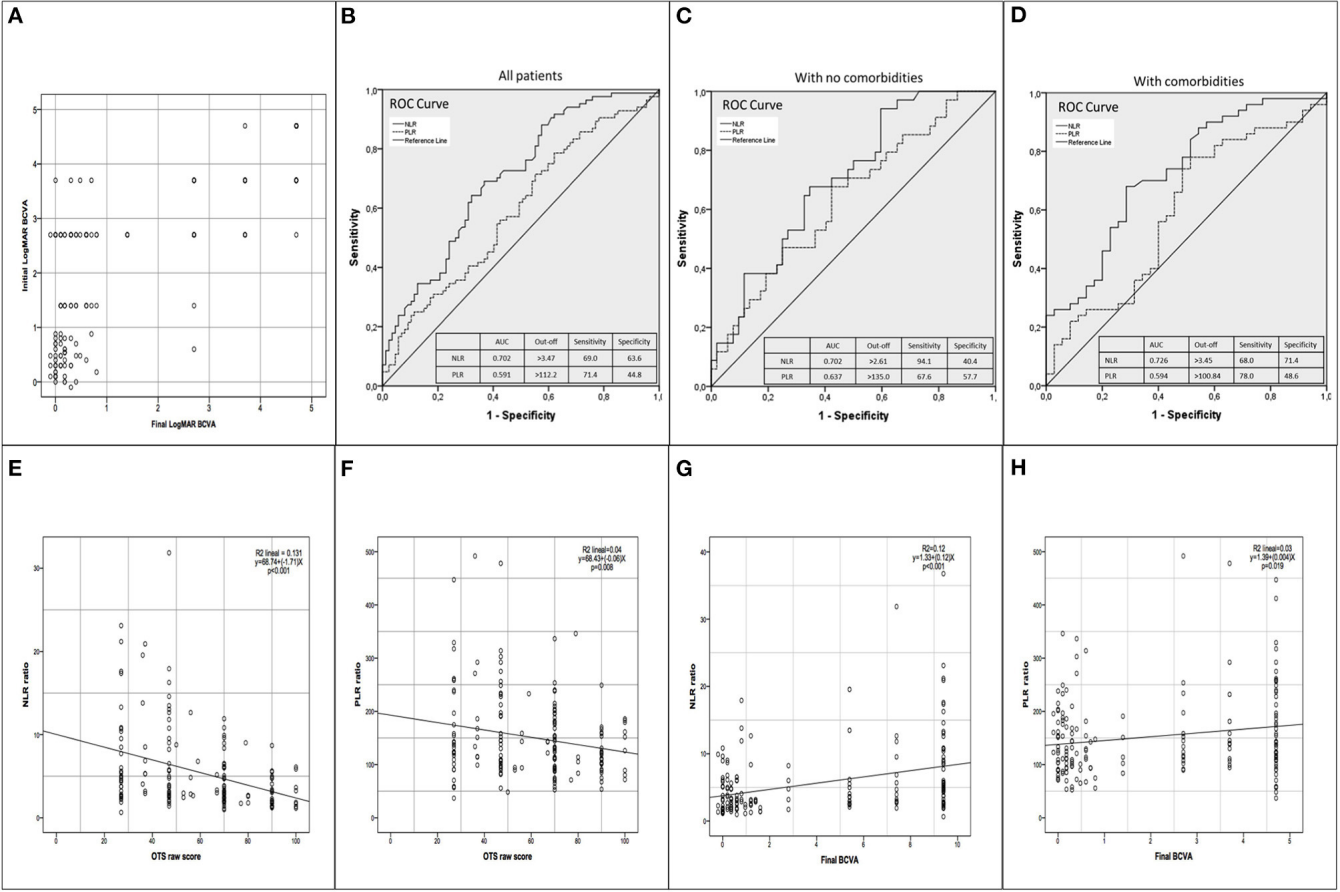
**(A)** Scatterplot of the initial LogMAR best corrected visual acuity (BCVA) with the final LogMAR BCVA. Patients with better initial BCVA were more likely to have better final BCVA. The majority that ended with severe visual impairment (SVI) had poor initial BCVA (LogMAR >2.00 equivalent to count fingers or worst). Only 2 patients with initial BCVA better than count fingers ended with SVI. **(B)** Receiver Operating Characteristics (ROC) curve analysis for discrimination between patients with SVI and Non-SVI (NSVI) in all patients. **(C)** ROC curve analysis for discrimination between patients with SVI and NSVI only in patients with no comorbidities. **(D)** ROC curve analysis for discrimination between patients with SVI and NSVI only in patients with comorbidities. **(E)** Scatterplot and linear regression of neutrophil to lymphocyte ratio (NLR) with the ocular trauma score (OTS) raw points. **(F)** Scatterplot and linear regression of platelet to lymphocyte ratio (PLR) with the OTS raw points. **(G)** Scatterplot and linear regression of NLR with the final BCVA. **(H)** Scatterplot and linear regression of PLR with the final BCVA.

A comparison of the initial clinical presentation of the OGI between patients with SVI and NSVI is shown in Table 2. The patients with SVI had significantly lower raw OTS, worse initial BCVA, larger wounds, and more sutures. Furthermore, an initial BCVA 20/200 or worse was significantly associated with patients with the SVI at the last visit. An initial BCVA 20/200 or worse was present in 98.9% of patients that ended with SVI vs. 49.5% of patients that ended with NSVI (*p* < 0.001). Of the 142 patients with initial BCVA 20/200 or worse, 92 (65%) ended with SVI. On the contrary, of the 52 patients with initial BCVA better than 20/200, only 1 (2%) ended with SVI (Figure 1A). The patients with SVI showed a significantly higher prevalence of globe rupture, zone 3 injury, total corneal injury, uveal exposure, vitreous exposure, athalamia, hyphema, and anterior chamber fibrin. Whereas, patients with NSVI presented more frequently with penetrating wounds, intraocular foreign body, zone 1 injury, and paracentral corneal injury (Table 2).

**Table 2:** Clinical characteristics of the OGIs in SVI and NSVI patients.

**Characteristics**	**All**	**SVI**	**NSVI**	***P*=**
	**(*n* = 197)**	**(*n* = 96)**	**(*n* = 101)**	
Ocular trauma score (OTS) raw points, mean ± SD	60.0 ± 23.2	41.5 ± 14.6	77.0 ± 15.2	**<0.001**
Initial BCVA (LogMAR), mean ± SD	2.6 ± 1.6	3.8 ± 0.9	1.4 ± 1.1	**<0.001**
Initial BCVA 20/200 or worse	142 (73.2)	92 (98.9)	50 (49.5)	**<0.001**
Final BCVA (LogMAR), mean ± SD	2.1 ± 2.0	4.0 ± 1.0	0.2 ± 0.2	**<0.001**
Wound length, millimeter, mean ± SD	16.8 ± 28.0	25.9 ± 36.4	11.2 ± 19.3	**<0.001**
Number of sutures, mean ± SD	8.3 ± 6.0	13.6 ± 6.5	5.9 ± 3.9	**<0.001**
**Type of injury**				
Globe rupture (*n*, %)	89 (45.2)	76 (79.2)	13 (12.9)	**<0.001**
Penetrating wound (*n*, %)	78 (39.6)	12 (12.5)	66 (65.3)	**<0.001**
Intraocular foreign body (*n*, %)	24 (12.2)	5 (5.2)	19 (18.8)	**0.004**
Perforating wound (*n*, %)	6 (3.0)	3 (3.1)	3 (3.0)	1.000
**Zones of injury**				
Zone 1 (Cornea and limbus) (*n*, %)	87 (50.6)	20 (28.2)	67 (66.3)	**<0.001**
Zone 2 (<5 mm from limbus) (*n*, %)	42 (24.4)	15 (21.1)	27 (26.7)	0.472
Zone 3 (>5 mm from limbus) (*n*, %)	43 (25)	36 (50.7)	7 (6.9)	**<0.001**
Wounds with corneal injury	137 (69.5)	46 (47.9)	91 (90.9)	**<0.001**
Central corneal (*n*, %)	21 (15.3)	4 (8.7)	17 (18.7)	0.141
Paracentral corneal (*n*, %)	35 (25.5)	2 (4.3)	33 (36.3)	**<0.001**
Peripheral corneal (*n*, %)	42 (30.7)	19 (41.3)	23 (25.3)	0.077
Total corneal (*n*, %)	39 (28.5)	21 (45.7)	18 (19.8)	**0.002**
Siedel + (*n*, %)	92 (56.1)	43 (62.3)	49 (51.6)	0.203
Uveal exposure (*n*, %)	120 (64.9)	74 (88.1)	46 (45.5)	**<0.001**
Vitreous exposure (*n*, %)	51 (29.8)	36 (50.7)	15 (15.0)	**<0.001**
Athalamia (*n*, %)	68 (40.0)	51 (71.8)	17 (17.2)	**<0.001**
Hyphema (*n*, %)	94 (52.5)	65 (82.3)	29 (29.0)	**<0.001**
Anterior chamber fibrin (*n*, %)	51 (34.0)	23 (45.1)	28 (28.3)	**0.046**
Hypopyon (*n*, %)	7 (4.6)	0 (0.0)	7 (7.0)	0.096
Traumatic cataract (*n*, %)	70 (53.4)	19 (55.9)	51 (52.6)	0.842
Anterior capsule rupture (*n*, %)	42 (35.9)	9 (37.5)	33 (35.5)	1.000
Initial retinal detachment (*n*, %)	6 (8.5)	1 (9.1)	5 (8.3)	1.000

*SVI, Severe visual impairment; NSVI, Non-SVI; OGI, Open globe injury; SD, standard deviation*.

*Bold means p < 0.05. Percentages at each parameter are relative to the number of patients with available data*.

Twenty-five (12.7%) patients had blood tests performed elsewhere and were excluded from the analysis of blood test parameters because these were not available. The remaining 172 (87.3%) patients had blood tests performed in our hospital and were included for analysis. The differences in blood test parameters between patients with SVI and NSVI are shown in Table 2. The patients with SVI had significantly higher LEU and NEU counts, a correspondent lower LYM count, greater NLR and PLR ratios, and higher glucose levels. These significant differences were observed when analyzing all patients together and when separated in patients with comorbidities and without comorbidities, except for PLR that was not significant in patients with comorbidities (Table 3). The clinical characteristics of OGI at presentation and its relationship with NLR and PLR ratios are shown in Table 3. Patients presenting with globe rupture, zone 3 injury, uveal exposure, vitreous exposure, hyphema or retinal detachment had significantly higher NLR and PLR. Patients presenting with total corneal injury had significantly higher NLR. Whereas, those presenting with intraocular foreign body, zone 1 injury, paracentral corneal injury, or hypopyon had significantly lower NLR and PLR, and patients with penetrating wounds had significantly lower NLR (Table 4). When analyzing only patients without comorbidities, the same result was observed except for total corneal injury. Likewise, when analyzing patients with comorbidities, the same was found, except for globe rupture, intraocular foreign body, paracentral and total corneal injury, vitreous exposure, and hypopyon (Table 4).

**Table 3:** Blood test parameters in SVI and NSVI patients with and without comorbidities.

**Parameter (Mean ± SD)**	**All patients (*****n*** **=** **172)**			**Without comorbidities (*****n*** **=** **87)**			**With comorbidities (*****n*** **=** **85)**		
	**SVI**	**NSVI**	***P*=**	**SVI**	**NSVI**	***P*=**	**SVI**	**NSVI**	***P*=**
	**(*n* = 84)**	**(*n* = 88)**		**(*n* = 34)**	**(*n* = 53)**		**(*n* = 50)**	**(*n* = 35)**	
Accident-blood sample interval (hours)	44.4 ± 76	49.2 ± 92	0.731	57.0 ± 100	39.1 ± 55	0.363	33.1 ± 42	64.8 ± 131	0.171
Hemoglobin (g/dL)	15.0 ± 1.8	15.3 ± 1.2	0.177	15.1 ± 1.8	15.3 ± 1.3	0.408	14.9 ± 1.8	15.2 ± 0.9	0.309
Hematocrit (%)	45.0 ± 5.0	45.9 ± 3.4	0.136	45.1 ± 4.4	46.2 ± 3.8	0.243	44.9 ± 5.5	45.6 ± 2.8	0.485
Leucocytes (K/uL)	11.7 ± 4.0	9.4 ± 3.0	**<0.001**	11.5 ± 3.6	9.3 ± 2.7	**0.001**	11.9 ± 4.2	9.6 ± 3.3	**0.002**
Neutrophils (K/uL)	9.2 ± 4.1	6.7 ± 3.0	**<0.001**	9.2 ± 3.9	6.7 ± 2.9	**0.002**	9.2 ± 4.3	6.6 ± 3.1	**0.001**
Lymphocytes (K/uL)	1.7 ± 0.9	2.1 ± 0.9	**0.014**	1.5 ± 0.5	1.9 ± 0.8	**0.006**	1.8 ± 1.0	2.3 ± 0.9	**0.011**
Platelets (K/uL)	249.0 ± 96	244.7 ± 54	0.709	249.7 ± 61	241.4 ± 48	0.495	249 ± 115	249.8 ± 62	0.954
NLR	7.4 ± 6.6	4.0 ± 3.2	**<0.001**	7.7 ± 6.6	4.4 ± 3.5	**0.001**	7.2 ± 6.7	3.5 ± 2.6	**<0.001**
PLR	167.2 ± 92	139.0 ± 64	**0.021**	189 ± 100	146.0 ± 63	**0.018**	152.7 ± 85	128.5 ± 65	0.160
MXD	0.8 ± 0.4	0.8 ± 0.3	0.304	0.7 ± 0.3	0.7 ± 0.3	0.830	0.9 ± 0.4	0.8 ± 0.4	0.508
Glucose (mg/dL)	124.8 ± 50	106.5 ± 35	**<0.001**	115.1 ± 36	99.0 ± 15	0.070	130.2 ± 56	117.4 ± 51	**0.019**

*SVI, Severe visual impairment; NSVI, Non-SVI; OGI, Open globe injury; SD, Standard deviation; NLR, Neutrophil-to-lymphocyte ratio; PLR, Platelet-to-lymphocyte ratio*.

*Bold means p < 0.05*.

**Table 4:** Clinical characteristics of OGI and its relationship with NLR and PLR.

**Characteristics**		**All patients (*****n*** **=** **172)**			**Without comorbidities (*****n*** **=** **87)**			**With comorbidities (*****n*** **=** **85)**		
		*n* ^*^	**NLR**	**PLR**	*n* ^*^	**NLR**	**PLR**	*n* ^*^	**NLR**	**PLR**
			**mean ± SD**	**mean ± SD**		**mean ± SD**	**mean ± SD**		**mean ± SD**	**mean ± SD**
Globe rupture	No	91	4.4 ± 4.4	139 ± 64	51	3.7 ± 2.4	136 ± 53	40	5.2 ± 6.0	142 ± 77
	Yes	81	7.2 ± 6.0	169 ± 93	36	8.5 ± 6.5	202 ± 101	45	6.1 ± 5.3	143 ± 79
	*P*=		**<0.001**	**0.015**		**<0.001**	**<0.001**		0.442	0.959
Penetrating wound	No	107	6.6 ± 5.6	159 ± 88	52	7.0 ± 6.1	176 ± 94	55	6.1 ± 5.0	143 ± 80
	Yes	65	4.3 ± 4.9	144 ± 66	35	3.7 ± 2.4	145 ± 57	30	5.0 ± 6.7	142 ± 76
	*P*=		**<0.001**	0.205		**0.002**	0.061		**0.032**	0.976
Intraocular foreign body	No	149	5.8 ± 5.7	157 ± 82	71	6.1 ± 5.6	174 ± 86	78	5.7 ± 5.8	142 ± 76
	Yes	23	4.3 ± 2.7	127 ± 63	16	3.8 ± 2.5	118 ± 36	7	5.5 ± 3.1	146 ± 103
	*P*=		**0.038**	**0.047**		**0.014**	**0.005**		0.939	0.904
Zone 1 injury	No	74	7.2 ± 6.2	183 ± 93	41	7.8 ± 6.4	200 ± 95	33	6.4 ± 5.9	160 ± 87
	Yes	75	3.7 ± 2.5	124 ± 49	44	3.7 ± 2.6	126 ± 44	31	3.7 ± 2.3	121 ± 56
	*P*=		**<0.001**	**<0.001**		**<0.001**	**<0.001**		**0.018**	**0.039**
Zone 2 injury	No	113	5.5 ± 5.0	151 ± 82	67	5.7 ± 5.5	157 ± 85	46	5.4 ± 4.5	142 ± 78
	Yes	36	5.0 ± 4.7	161 ± 73	18	5.6 ± 4.2	182 ± 70	18	4.4 ± 5.2	141 ± 72
	*P*=		0.552	0.481		0.916	0.220		0.490	0.953
Zone 3 injury	No	111	4.1 ± 3.4	136 ± 60	62	4.2 ± 3.2	142 ± 58	49	4.0 ± 3.6	129 ± 63
	Yes	38	9.2 ± 6.8	203 ± 106	23	9.5 ± 7.4	216 ± 110	15	8.8 ± 5.9	184 ± 100
	*P*=		**<0.001**	**<0.001**		**<0.001**	**0.001**		**0.001**	**0.023**
Central corneal injury	No	104	4.4 ± 3.1	139 ± 56	56	4.3 ± 2.9	137 ± 50	48	4.5 ± 3.2	142 ± 64
	Yes	16	4.4 ± 3.1	123 ± 63	9	4.4 ± 3.2	147 ± 70	7	4.3 ± 3.1	95 ± 44
	*P*=		0.947	0.358		0.947	0.708		0.873	0.071
Paracentral corneal injury	No	90	4.8 ± 3.2	144 ± 59	44	4.9 ± 3.1	149 ± 56	46	4.8 ± 3.3	140 ± 62
	Yes	30	3.1 ± 2.2	114 ± 45	21	3.2 ± 2.1	114 ± 33	9	3.0 ± 2.6	113 ± 69
	*P*=		**0.002**	**0.005**		**0.013**	**0.003**		**0.032**	0.254
Peripheral corneal injury	No	81	4.4 ± 3.2	133 ± 56	49	4.3 ± 3.0	137 ± 52	32	4.7 ± 3.6	126 ± 61
	Yes	39	4.3 ± 2.9	146 ± 60	16	4.5 ± 2.9	141 ± 52	23	4.2 ± 2.9	149 ± 65
	*P*=		0.860	0.252		0.789	0.767		0.601	0.195
Total corneal injury	No	85	3.9 ± 2.7	130 ± 57	46	3.9 ± 2.7	130 ± 49	39	4.0 ± 2.9	131 ± 65
	Yes	35	5.6 ± 3.6	152 ± 56	19	5.4 ± 3.3	157 ± 55	16	5.8 ± 3.9	147 ± 58
	*P*=		**0.015**	0.053		0.054	0.051		0.053	0.403
Positive Seidel test	No	59	5.0 ± 4.3	147 ± 70	36	4.5 ± 3.2	147 ± 57	23	5.8 ± 5.7	146 ± 88
	Yes	85	5.6 ± 5.2	156 ± 88	43	6.5 ± 6.4	175 ± 103	42	4.8 ± 3.5	138 ± 66
	*P*=		0.408	0.476		0.077	0.142		0.458	0.690
Uveal exposure	No	57	3.8 ± 2.9	127 ± 56	36	3.9 ± 2.5	137 ± 53	21	3.7 ± 3.5	110 ± 58
	Yes	104	6.6 ± 6.2	166 ± 84	50	7.1 ± 6.2	182 ± 94	54	6.2 ± 6.2	151 ± 72
	*P*=		**<0.001**	**0.001**		**0.009**	**0.025**		**0.006**	**0.004**
Vitreous exposureo	No	100	4.5 ± 4.0	136 ± 60	55	4.4 ± 3.8	141 ± 59	45	4.6 ± 4.4	131 ± 60
	Yes	50	7.4 ± 6.0	185 ± 95	28	8.6 ± 6.7	210 ± 104	22	5.8 ± 4.7	152 ± 72
	*P*=		**<0.001**	**0.001**		**<0.001**	**0.001**		0.285	0.206
Athalamia	No	87	4.5 ± 3.4	145 ± 62	54	4.5 ± 3.4	146 ± 58	33	4.4 ±3.4	143 ± 68
	Yes	62	5.5 ± 5.1	151 ± 94	24	6.7 ± 6.7	184 ± 113	38	4.8 ± 3.7	131 ± 73
	*P*=		0.163	0.625		0.059	0.052		0.671	0.464
Hyphema	No	73	3.4 ± 2.3	130 ± 58	43	3.5 ± 2.3	132 ± 51	30	3.3 ± 2.3	128 ± 68
	Yes	83	6.9 ± 5.7	165 ± 88	39	7.2 ± 6.3	186 ± 95	44	6.6 ± 5.3	146 ± 77
	*P*=		**<0.001**	**0.004**		**<0.001**	**0.001**		**<0.001**	0.307
Anterior chamber fibrin	No	91	4.8 ± 4.8	143 ± 80	53	5.2 ± 5.4	152 ± 89	38	4.3 ± 3.7	130 ± 65
	Yes	41	4.9 ± 2.8	154 ± 50	20	4.7 ± 2.8	155 ± 45	21	5.1 ± 2.9	152 ± 56
	*P*=		0.066	0.345		0.601	0.841		**0.029**	0.192
Hypopyon	No	128	4.9 ± 4.3	148 ± 72	69	5.2 ± 4.9	157 ± 80	59	4.6 ± 3.4	138 ± 62
	Yes	6	3.0 ± 0.6	108 ± 29	5	3.1 ± 0.7	112 ± 31	1	2.4	90
	*P*=		**<0.001**	**0.019**		**0.002**	**0.029**		0.537	0.444
Traumatic cataract	No	53	4.2 ± 2.9	138 ± 65	28	4.9 ± 3.4	152 ± 68	25	3.3 ± 2.0	122 ± 59
	Yes	61	4.3 ± 3.6	145 ± 70	39	4.3 ± 3.9	148 ± 75	22	4.2 ± 3.0	141 ± 62
	*P*=		0.858	0.539		0.483	0.813		0.211	0.283
Anterior capsule rupture	No	67	4.2 ± 3.2	147 ± 73	36	4.8 ± 3.9	158 ± 80	31	3.6 ± 2.1	134 ± 63
	Yes	34	4.2 ± 3.7	135 ± 59	23	4.1 ± 3.8	135 ± 58	11	4.4 ± 3.8	136 ± 63
	*P*=		0.952	0.403		0.514	0.207		0.414	0.911
Retinal detachment	No	52	3.5 ± 2.9	133 ± 57	34	4.1 ± 3.4	147 ± 62	18	2.4 ± 1.3	107 ± 38
	Yes	5	10.2 ± 6.8	286 ± 148	3	13.4 ± 6.4	332 ± 140	2	5.5 ± 5.0	218 ± 182
	*P*=		**0.015**	**0.018**		**0.004**	**0.006**		**0.028**	**0.017**

*OG0, Open globe injury; SVI, Severe visual impairment; NSVI, Non-SVI; SD, Standard deviation; NLR, Neutrophil-to-lymphocyte ratio; PLR, Platelet-to-lymphocyte ratio*.

* *Number of patients with available data*.

*Bold means p < 0.05*.

The correlations of NLR and PLR ratios with continuous variables are shown in Table 4. NLR and PLR had a significant negative correlation with raw OTS and accident-blood sample interval, a significant positive correlation with initial LogMAR BCVA, and the number of sutures. NLR had a significant positive correlation with final LogMAR BCVA and wound length. In general, NLR showed stronger and more consistent correlations than PLR. NLR and PLR had stronger correlations in patients without comorbidities. PLR showed no significant differences in patients with comorbidities (Table 5). The univariate logistic regression analysis found many variables had an increased risk for SVI. A detailed description is shown in Table 5, those with OR > 5 were polytrauma (OR = 5.80), trauma with fist/kick (OR = 7.35), blunt objects (OR = 12.07), globe rupture (OR = 25.72), zone 3 injury (OR = 13.81), uveal exposure (OR = 8.84), vitreous exposure (OR = 5.83), hyphema (OR = 11.37), and athalamia (OR = 12.30). Also, the risk to end with SVI showed a 4-fold increase when NLR ≥ 3.47 (OR = 3.90) and a 2-fold increase when PLR ≥ 112.2 (OR = 2.03) (Table 6). However, when included in the multivariate analysis only athalamia [OR = 3.75, (1.04–13.45), *p* = 0.042], hyphema [OR = 4.92 (1.38–17.45), *p* = 0.014] and lower OTS [OR = 1.09 (1.04–1.14), *p* < 0.0001] retained its statistical significance as risk factor for SVI.

**Table 5:** Spearman's correlation coefficient between NLR and PLR and the studied variables.

	**All (*****n*** **=** **172)**				**Without comorbidities (*****n*** **=** **87)**				**With comorbidities (*****n*** **=** **85)**			
	**NLR**		**PLR**		**NLR**		**PLR**		**NLR**		**PLR**	
	**σ**	***P***	**σ**	***P***	**σ**	***P***	**σ**	***P***	**σ**	***P***	**σ**	***P***
OTS, raw points	−0.389	**<0.001**	−0.166	**0.031**	−0.562	**<0.001**	−0.442	**<0.001**	−0.246	**0.026**	-0.009	0.936
Initial BCVA (LogMAR)	0.392	**<0.001**	0.157	**0.042**	0.477	**<0.001**	0.349	**0.001**	0.334	**0.002**	0.055	0.624
Final BCVA (LogMAR)	0.345	**<0.001**	0.123	0.108	0.383	**<0.001**	0.283	**0.008**	0.364	**0.001**	0.077	0.481
Wound length, (millimeter)	0.173	**0.046**	0.091	0.301	0.261	**0.025**	0.180	0.127	0.100	0.451	0.043	0.744
Number of sutures	0.379	**<0.001**	0.260	**0.004**	0.444	**<0.001**	0.390	**0.001**	0.361	**0.009**	0.193	0.169
Accident-blood sample interval	−0.392	**<0.001**	−0.286	**0.001**	−0.451	**<0.001**	−0.338	**0.003**	−0.334	**0.007**	-0.245	0.051

*OTS, Ocular trauma score; OGI, Open globe injury; NLR, Neutrophil-to-lymphocyte ratio; PLR, Platelet-to-lymphocyte ratio; BCVA, Best corrected visual acuity*.

*Correlation analysis with Spearman's correlation coefficient*.

*Bold means p < 0.05*.

**Table 6:** Univariate logistic regression analysis for severe visual impairment.

**Variable**	***n* (%)**	**OR**	**C.I. 95%**	***P*=**
Gender, men	86 (89.6)	1.06	0.42–2.67	0.905
Age (mean ± SD) years	39.5 ± 15.4	1.021	1.00–1.04	**0.044**
Follow-up days (median, IQR)	30, 80	1.00	0.99–1.00	0.685
**Systemic comorbidities**				
Illegal substance abuse	12 (14.0)	3.73	1.16–12.04	**0.028**
Hypertension	10 (11.6)	3.06	0.92–10.14	0.067
Diabetes	5 (5.8)	1.93	0.45–8.34	0.376
**Mechanism of injury**				
Metallic object trauma	18 (18.8)	0.18	0.96–0.35	**<0.001**
Polytrauma	14 (14.6)	5.80	1.61–20.92	**0.007**
Fist/kick trauma	12 (12.5)	7.35	1.60–33.80	**0.010**
Blunt objects trauma	12 (12.5)	12.07	1.51–96.29	**0.019**
Explosives trauma	4 (4.2)	–	–	0.999
OTS raw points (mean ± SD)	41.5 ± 14.6	1.138	1.10–1.18	**<0.001**
Wound length, millimeters (mean ± SD)	25.9 ± 36.4	1.022	1.01–1.04	**0.007**
Number of sutures	13.6 ± 6.5	1.333	1.21–1.47	**<0.001**
**Type and zones of injury**				
Penetrating wound	12 (12.5)	0.76	0.36–0.16	**<0.001**
Globe rupture	76 (79.2)	25.72	11.99–55.15	**<0.001**
Zone 3 trauma (>5 mm from limbus)	36 (50.7)	13.81	5.62–33.89	**<0.001**
Peripheral corneal injury	19 (41.3)	2.08	0.98–4.42	0.057
Total corneal injury	21 (45.7)	3.41	1.57–7.40	**0.002**
**Clinical characteristics of OGI**				
Intraocular foreign body	5 (5.2)	0.24	0.85–0.66	**0.006**
Uveal exposure	74 (88.1)	8.84	4.10–19.06	**<0.001**
Vitreous exposure	36 (50.7)	5.83	2.83–11.96	**<0.001**
Hyphema	65 (82.3)	11.37	5.52–23.38	**<0.001**
Athalamia	51 (71.8)	12.30	5.89–25.65	**<0.001**
Anterior chamber fibrin	23 (45.1)	2.08	1.03–4.21	**0.041**
Initial retinal detachment	1 (9.1)	1.10	0.12–10.43	0.934
**Blood test parameters**				
Neutrophil to lymphocyte ratio (NLR) ≥ 3.47	58 (64.4)	3.90	2.07–7.36	**<0.001**
Platelet to lymphocyte ratio (PLR) ≥ 112.2	60 (55.6)	2.03	1.08–3.83	**0.029**
Absolute leucocyte count (mean ± SD)	11.7 ± 3.9	1.23	1.11–1.37	**<0.001**
Absolute neutrophil count (mean ± SD)	9.2 ± 4.1	1.24	1.12–1.37	**<0.001**
Absolute lymphocyte count (mean ± SD)	1.7 ± 0.9	0.59	0.39–0.86	**0.008**
Glucose (mean ± SD)	124.8 ± 50	1.01	1.00–1.02	**0.027**

*OTS, Ocular trauma score; BCVA, best corrected visual acuity; IQR, Interquartile range; SVI, Severe visual impairment; NSVI, Non-SVI; OGI, Open globe injury*.

*Bold means p < 0.05*.

*Percentages are relative to the number of patients with available data*.

The ROC analysis of NLR and PLR for SVI and NSVI is shown in Figures 1B–D. In all patients, the area under the ROC (AUROC) value for NLR and PLR that distinguish between SVI and NSVI was 0.702 (CI 0.624–0.779, *p* < 0.001) and 0.591 (CI 0.506–0.676, *p* = 0.040) respectively. The best cut-off value for NLR was 3.47 (sensitivity of 69.0% and a specificity of 63.6%) and for PLR 112.2 (sensitivity of 71.4% and a specificity of 44.8%). The positive predictive value (PPV) and the negative predictive value (NPV) for NLR were 64.4 and 68.3%, respectively. For PLR, the PPV and NPV were 55.6 and 61.9%, respectively. Furthermore, the NLR ≥ 3.47 and PLR ≥ 112.2 together had a sensitivity of 57.1%, specificity of 65.9%, PPV of 61.5%, and NPV of 61.7%, for distinguishing between SVI and NSVI. The relationship between NLR and PLR with OTS is shown in Figures 1E,F. Patients with higher pre-operative NLR or PLR were more likely to have lower OTS. The relationship between NLR and PLR with final LogMAR BCVA is shown in Figures 1G,H. Patients with higher pre-operative NLR or PLR were more likely to have greater LogMAR BCVA; a greater LogMAR value means worse vision.

## Discussion

Almost half of the patients ended with SVI after OGI repair. To the best of our knowledge, this study shows for the first time that patients with repaired OGI that ended with SVI had increased pre-operative NLR and PLR levels compared to patients that achieved a better vision. NLR showed a positive correlation with final LogMAR BCVA in all patients with or without comorbidities, but this was stronger in patients without comorbidities, which means that the higher the pre-operative NLR, the worse the final visual acuity. PLR showed a positive correlation with final LogMAR BCVA only in patients without comorbidities. The correlation of PLR in patients with comorbidities was absent. NLR and PLR were considered risk factors for SVI after OGI repair in a univariate analysis. Furthermore, a known prognostic score for poor final BCVA after OGI, the OTS, showed a negative correlation with NLR and PLR, which means that higher NLR and PLR were associated with lower OTS and poor final BVCA in this study. Even more, ROC analysis found that NLR ≥ 3.47 and PLR ≥ 112.2 were the best cut-off values to predict SVI after OGI repair. The risk to develop SVI showed a 4-fold increase when NLR ≥ 3.47 and a 2-fold increase when PLR ≥ 112.2 in univariate analysis. However, on multivariate analysis, only athalamia, hyphema, uveal exposure, and lower OTS remained as risk factors for SVI after OGI repair. In addition, we evaluated demographics characteristics, mechanism of injury, OTS, initial BCVA, wound characteristics, and blood test parameters to look for associations with high NLR, high PLR, and risk factors for SVI after OGI repair.

In OGI the natural ocular barriers have been trespassed, and appropriate control of immune tolerance and regulation may not be achieved, resulting in increased local and systemic inflammation, and damage. However, it is not clear if OGI has a systemic inflammatory effect. The NLR and the PLR are non-specific parameters for systemic inflammation. This is the first study to describe its relationship with OGI and final visual acuity. We believe that traumatic ocular damage can lead to a systemic inflammatory response, which can be detected by NLR and PLR.

Higher LEU, NEU and glucose, and lower LYM counts were all associated with SVI in univariate analysis. The fact that the NLR, PLR, and NEU levels were elevated and LYM levels were reduced indicating a systemic inflammation with poor regulation (15). In this study, OGI presenting with globe rupture, zone 3 injury, uveal exposure, vitreous exposure, hyphema or retinal detachment, had higher NLR and PLR. These clinical characteristics were associated with increased damage of globe structure and worse visual outcome, in previous studies (4, 5, 7, 8).

As previously mentioned, a proportional correlation between NLR and PLR with initial and final LogMAR visual acuity was observed. That means that the higher the NLR and PLR, the worse the initial and final BCVA. However, the majority of these were weak correlations (*r* < 0.4), the stronger and more significant correlations were observed when analyzing the NLR or the group without comorbidities where moderate correlations (*r* = 4–6) of NLR with OTS, NLR with initial BCVA, and PLR with OTS were found. High pre-operative NLR or PLR could be considered as an aid in the identification of patients with a higher risk for SVI before the OGI is repaired. This can be helpful to identify patients that might benefit from pre-operative counseling about their poor visual prognosis. In agreement with that, as the levels of NLR and PLR were increased, the raw OTS was reduced, indicating a worse OTS category and a worse prognosis for final visual acuity, as observed in Figures 1E–H. Furthermore, the risk to develop SVI showed a 4-fold increase when NLR ≥ 3.47, a 2-fold increase when PLR ≥ 112.2. The univariate logistic regression analysis demonstrates that NLR, and in a lesser manner the PLR are directly related to the severity of trauma and with the final visual prognosis after OGI repair. These findings also confirm the role of the OTS as a great prognostic tool in OGI (Table 6). However, the OTS oftentimes cannot be performed adequately because of missing information like ultrasound evaluation, retinal detachment, and RAPD. In these cases, the NLR and PLR have the advantage that can be used as a complementary index to assess visual prognosis when not all the variables required to perform the OTS are available. It can be performed easily before or after OGI repair because it only requires standard pre-operative blood tests.

A higher prevalence of initial BCVA 20/200 or worse was found in patients with final SVI. Two-thirds of patients with initial BCVA 20/200 or worse ended with SVI, on the contrary, only 2% of those with initial BCVA better than 20/200, ended with SVI (Figure 1A). Other parameters found to be associated with SVI were globe rupture, zone 3 injuries, longer wounds with a greater number of sutures, total corneal injury, uveal exposure, vitreous exposure, athalamia, hyphema, and anterior chamber fibrin. These clinical findings are similar to those found in other studies (43–46). Okamoto et al. recently described worse initial and final BCVA in ruptured globes in comparison with laceration and also found that greater wound length was significantly correlated with worse final BCVA (43). The results found in the present study correlate with previous studies that described the presence of hyphema, uveal and vitreous exposure at initial presentation to be associated with poor VA and ocular prognosis (44–46). The initial BCVA was significantly worse in patients with SVI, which is in agreement with previous publications that consider it as a risk factor for poor final visual outcome (43–50). Older age was also associated with poor final VA and is in agreement with Agrawal et al. that found worse final visual outcome with increasing age (51). This could be due to reduced wound healing potential, decreased corneal and scleral collagen crimp (52), increase in non-enzymatic crosslinking, decrease in hydration stability, and glycosaminoglycans, and more overall globe stiffness (53). The mechanism of injury more frequently observed in patients with SVI were polytrauma, fist/kick, blunt object, and explosives. They all share in common a high probability of producing a contuse blunt blow trauma instead of a puncture or cut and consequently a globe rupture injury.

On the other hand, more than half of the patients with NSVI had an OGI that was generated with a metallic object that resulted in a penetrating wound that involved zone 1 or paracentral cornea. Penetrating, metallic object trauma had reduced risk for SVI in univariate analysis. Penetrating wounds, zone 1 and paracentral corneal injury had significantly lower NLR. The patients with NSVI had longer median follow-up, this might be because they had better visual acuity and required closer and longer follow-up to take care of the eye and visual rehabilitation. On the contrary, those patients with SVI showed lower median follow-up probably because they needed fewer follow-up examinations and only palliative care of the eye. The NLR and PLR showed a weak significant negative correlation with the accident-blood sample interval, which means that the NLR tends to decrease as more time passes between the accident and the collection of blood samples. In cases with longer accident-blood sample intervals, the sensibility of NLR or PLR as a predictor of poor final visual acuity might be reduced. However, this correlation was weak, and since the hypothesis of this study is that higher levels of NLR or PLR area are associated with SVI, this might not affect the specificity to detect those at high risk for SVI.

The strengths of this study are that it is the first to correlate the NLR and the PLR in acute ocular trauma. In addition, this study included all consecutive patients who arrived with an OGI. Analyzing patients separately with and without comorbidities addressed the bias that systemic comorbidities can have in the interpretation of the NLR and PLR. These parameters are already present in the report of the CBC and could be used in patients with OGI to aid in the assessment of the risk of ending with SVI.

We acknowledge several limitations in our study. Firstly that simultaneous trauma in the orbit, face, or other parts of the body could have influenced the laboratory findings and were not addressed in this study. We believe that the mechanisms of trauma and characteristics of the injuries could affect the inflammation parameters if the orbit, facial or other parts of the body were traumatized and consequently releasing higher levels of glucocorticoids, inflammatory, vasoactive, and chemotactic substances, capable of changing the NLR and the PLR in this population. In addition, the associations identified between laboratory findings and the OTS and other ocular parameters that mostly indicate the magnitude of the eye damage would not be expected to be present if the laboratory findings were caused by trauma in other parts of the body. However, the aim of this study was to use the NLR and the PLR in all cases. Given the conditions of consecutive enrollment of patients, all types of mechanisms for OGIs that were surgically repaired were included, only the minority were polytraumatized and those treated with primary evisceration or enucleation were excluded. A similar limitation lies in the heterogeneity of the patients in this case series of consecutive patients since we included all types of OGIs such as globe rupture, IOFB, penetrating and perforating wounds; and the type of OGI was not evenly distributed between SVI and NSVI groups. Further studies should evaluate the impact of NLR and PLR in predicting final SVI by type and zone of injury. Secondly, C-reactive protein (CRP) and globular sedimentation rate (GSR) tests were not analyzed, which are the most commonly used parameters of systemic inflammation. The reason for this is that these studies are not done routinely as part of the pre-operative evaluations of eye wounds; however, it is worthwhile to carry further studies with more systemic inflammatory markers to confirm our findings. Thirdly, in the univariate and multivariate logistic analysis, initial BCVA was not included, the reason for this is that our population included very heterogeneous initial visual acuities to use this regression analysis.

In conclusion, this study reports for the first time that NLR and PLR were correlated with visual outcomes after OGI repair. Higher NLR and PLR were correlated with low OTS and SVI after OGI repair. NLR ≥ 3.47 or PLR ≥ 112.2 increases the risk for SVI 4 and 2 folds, respectively. NLR and PLR could be used as prognostic biomarkers for SVI after OGI repair. These findings pave the pad to further investigate the role of NLR and PLR as a prognostic tool for final visual acuity after surgical repair of OGI.

## Data Availability Statement

The raw data supporting the conclusions of this article will be made available by the authors, without undue reservation.

## Ethics Statement

The studies involving human participants were reviewed and approved by Comité de Ética en Investigación, Hospital Universitario Dr. José E. González y Facultad de Medicina, Universidad Autónoma de Nuevo León, Avenida Francisco I. Madero Avenida Jose Eleuterio Gonzalez Gonzalitos Colonia Mitras Centro, C.P. 64460, monterrey, Nuevo León, México. The patients/participants provided their written informed consent to participate in this study.

## Author Contributions

KM-N: conceptualization, patient evaluation, formal analysis, investigation, methodology, project administration, supervision, roles/writing—original draft, writing—review and editing, data curation, supervision, and validation. AT-H: conceptualization, data curation, patient evaluation, formal analysis, investigation, methodology, roles/writing—original draft, writing—review and editing, data curation, supervision, and validation. JM-N: conceptualization, patient evaluation, formal analysis, investigation, methodology, supervision, validation, visualization, and review and editing. BV-S: formal analysis, investigation, software, roles/writing—original draft, and writing—review and editing. VM-P: investigation, data curation, formal analysis, patient evaluation, roles/writing—original draft, and writing—review and editing. DG-V: data curation, patient evaluation, and investigation. DR-M and AS-S: data curation. GV-M and JG-C: patient evaluation and review and editing. RE-O: supervision, validation, and review and editing. SG-L: supervision, validation, resources, and review and editing. JM-H: project administration, resources, investigation, methodology, supervision, validation, and review and editing. All authors contributed to the article and approved the submitted version.

## Funding

This project was funded with own resources of the Ophthalmology Department of the University Hospital and Faculty of Medicine of the Autonomous University of Nuevo Leon (UANL).

## Conflict of Interest

The authors declare that the research was conducted in the absence of any commercial or financial relationships that could be construed as a potential conflict of interest.

## Publisher's Note

All claims expressed in this article are solely those of the authors and do not necessarily represent those of their affiliated organizations, or those of the publisher, the editors and the reviewers. Any product that may be evaluated in this article, or claim that may be made by its manufacturer, is not guaranteed or endorsed by the publisher.
